# Anisotropic Thermal Expansion and Electronic Structure of LiInSe_2_

**DOI:** 10.3390/molecules27165078

**Published:** 2022-08-10

**Authors:** Victor V. Atuchin, Ludmila I. Isaenko, Sergei I. Lobanov, Alina A. Goloshumova, Maxim S. Molokeev, Zhaoming Zhang, Xingyu Zhang, Xingxing Jiang, Zheshuai Lin

**Affiliations:** 1 Laboratory of Optical Materials and Structures, Institute of Semiconductor Physics, SB RAS, Novosibirsk 630090, Russia; 2 Department of Applied Physics, Novosibirsk State University, Novosibirsk 630090, Russia; 3 Research and Development Department, Kemerovo State University, Kemerovo 650000, Russia; 4 Department of Industrial Machinery Design, Novosibirsk State Technical University, Novosibirsk 630073, Russia; 5 R&D Center “Advanced Electronic Technologies”, Tomsk State University, Tomsk 634034, Russia; 6 Laboratory of Crystal Growth, Sobolev Institute of Geology and Mineralogy, SB RAS, Novosibirsk 630090, Russia; 7 Laboratory of Functional Materials, Novosibirsk State University, Novosibirsk 630090, Russia; 8 Laboratory of Crystal Physics, Kirensky Institute of Physics, Federal Research Center KSC SB RAS, Krasnoyarsk 660036, Russia; 9 Institute of Engineering Physics and Radioelectronic, Siberian Federal University, Krasnoyarsk 660041, Russia; 10 Department of Physics, Far Eastern State Transport University, Khabarovsk 680021, Russia; 11 Australian Science and Technology Organisation (ANSTO), Lucas Heights, NSW 2234, Australia; 12 Functional Crystals Laboratory, Technical Institute of Physics and Chemistry, Chinese Academy of Sciences, Beijing 100190, China; 13 University of the Chinese Academy of Sciences, Beijing 100049, China

**Keywords:** LiInSe_2_, crystal growth, thermal expansion, band structure, XPS, DFT

## Abstract

Optical quality cm-sized LiInSe_2_ crystals were grown using the Bridgman–Stockbarger method, starting from pure element reagents, under the conditions of a low temperature gradient of 5–6 degrees/cm and a slight melt overheating. The phase purity of the grown crystal was verified by the powder XRD analysis. The thermophysical characteristics of LiInSe_2_ were determined by the XRD measurements in the temperature range of 303–703 K and strong anisotropy of the thermal expansion coefficients was established. The following values of thermal expansion coefficients were determined in LiInSe_2_: α_a_ = 8.1 (1), α_b_ = 16.1 (2) and α_c_ = 5.64 (6) MK^−1^. The electronic structure of LiInSe_2_ was measured by X-ray photoelectron spectroscopy. The band structure of LiInSe_2_ was calculated by ab initio methods.

## 1. Introduction

Complex chalcogenide compounds have interesting structural, chemical, and physical properties, and the materials are widely applied in modern optical and electronic technologies [1,2,3,4,5,6,7,8,9,10]. Among such compounds, the crystals with general composition LiMX_2_ (M = Al, In, Ga; X = S, Se, Te) have attracted particular research interests because of their valuable combination of optical and electronic characteristics, including thermal and chemical stabilities, wide transparency range, appropriate nonlinear optical coefficients, birefringence values and optical damage thresholds [2,11,12,13,14,15,16,17,18]. From the structural point of view, two main structure types are known for compounds LiMX_2_, as listed in Table 1 [19,20,21,22,23,24,25]: type I, space group *Pna*2_1_, is observed in sulfides and selenides and type II, space group *I*-42*d*, is common in tellurides. Moreover, the formation of trigonal high-temperature modification, space group *P*3*m*1, was detected for LiAlTe_2_ [25]. All these structures are without inversion center, and accordingly, the materials are characterized by valuable combinations of attractive physical properties [26]. As of now, the LiMX_2_ crystals are widely applied for the optical frequency conversion in visible-IR spectral ranges [2,15,27,28,29,30,31,32,33,34] and as a working medium in neutron detectors [35,36,37,38,39]. Accordingly, high-quality single crystals are urgently needed and the growth technology should be further developed to provide large-sized low-defect LiMX_2_ materials.

The thermal and thermophysical characteristics of LiMX_2_ materials are of great importance in the single crystal growth to avoid crystal cracking and defect generation [12,17,18,40,41,42]. However, such information is scarce in the literature and, in some cases, is doubtful. The thermophysical parameters earlier reported for LiMX_2_ crystals are summarized in Table 2 [43,44,45,46]. As it is seen, the behavior of type I and type II crystals is principally different, and in tellurides, the thermal expansion along crystallographic direction *c* is negative on heating. Comparatively, in sulfides and selenides, thermal expansion is positive in all crystallographic directions with increasing temperature. However, a detailed analysis of the thermophysical parameters is complicated by the following obstacles. As for the practically valuable LiInSe_2_, the thermal expansion coefficients were measured only in one study and a contradiction is found in the reported values [43]. The possible error ranges given in Figure 5 of [43] are above the integrated effect over the considered temperature range. At the same time, the possible error ranges reported for the numerical values of thermal expansion coefficients seem to be reasonable (Table 2). Moreover, the atomic mechanism of the thermal expansion in type I LiMX_2_ crystals has not been considered up to now. To avoid the uncertainty, the present work is aimed at the precise determination of the structural parameters of LiInSe_2_ on temperature using both experimental and theoretical methods, including a detailed analysis of thermal expansion mechanism. To attain this, a high-quality single crystal of LiInSe_2_ was grown by the developed technology and its structural parameters and electronic structure were determined. The electronic structure was observed in parallel by X-ray photoelectron spectroscopy (XPS) and theoretical methods, where XPS is extremely sensitive to the chemical state of the crystal surface and the theoretical model is a powerful tool to consider the relations between the crystal structure and physical properties.

## 2. Experimental Methods

**Figure 1:**
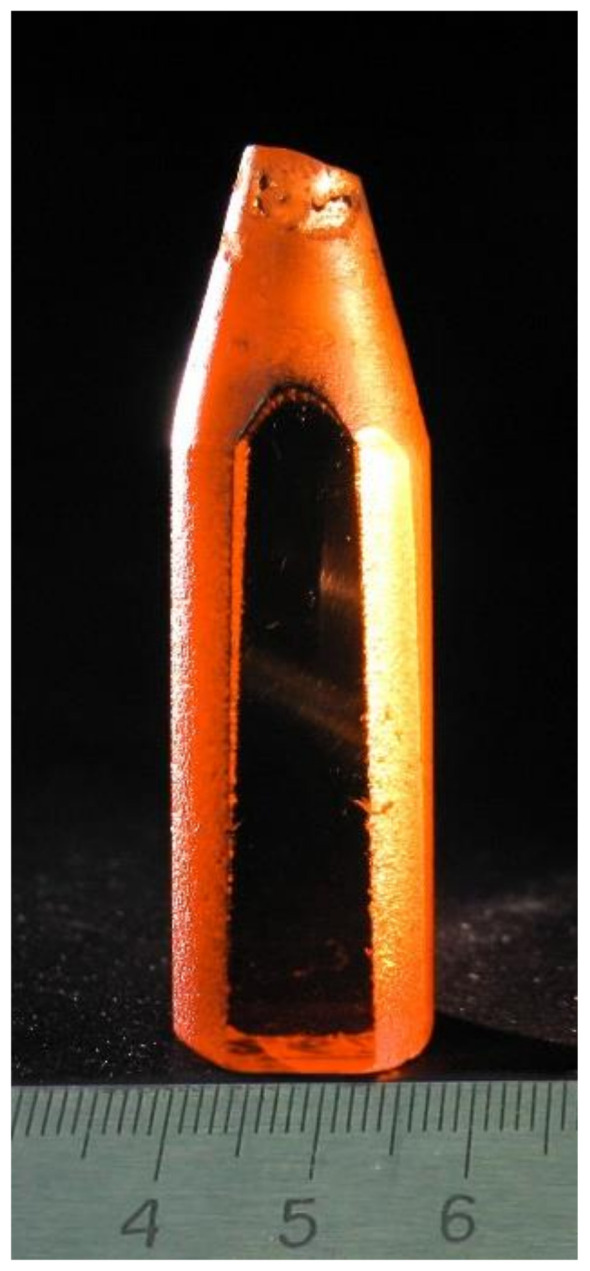
Digital image of the LiInSe_2_ crystal.

LiInSe_2_ crystals were grown using the Bridgman–Stockbarger method, as described in [12]. Li (3N, Novosibirsk Chemical Concentrates Plant, Novosibirsk, Russia), In (5N, Alfa Aesar GmbH, Haverill, MA, USA), and Se (5N, Reachem, Moscow, Russia) were used as starting materials. The melting point of the batch was about 915 °C. Since chalcogenide compounds contain volatile components and chemically aggressive lithium, the synthesis and growth were carried out in glass-graphite containers. In the process of chalcogenide compound synthesis and crystal growth, incongruent evaporation takes place at temperatures above the melting point [47,48,49,50]. As a result, there is a deviation of the composition from the stoichiometric one, but it can be reduced under the conditions of a low temperature gradient at the crystallization front and a small excess pressure in the ampoule. Deviation from stoichiometry is the cause of spot defects and changes in the LiInSe_2_ sample color from greenish and yellow to red with the variation of cell parameters [51]. In the first group, the crystal composition is close to stoichiometric composition with lithium and selenium vacancies (V_Li_, V_Se_). The presence of In_Li_ + 2V_Li_^−^ interatomic substitution with a charge compensator and interstitial Se_i_ atoms leads to an increase in red color intensity [52]. In this work, in contrast to the methodology described in [12], the crystals were grown under the conditions of a low gradient of 5–6 degrees/cm and a slight melt overheating (no more than 50 degrees above the melting point). The grown boules were annealed at 800 °C for 2 h. As a result, yellowish single crystals up to 17 mm in diameter 40 mm long of high optical quality were obtained (Figure 1). The applied method of crystal growth under the low thermal gradient conditions makes it possible to improve the crystal homogeneity along the ingot length and reduce the number of inclusions in the crystal volume. At the 5 cm^−1^ absorption level, the transparency range for the crystal is 0.47–13 μm.

The powder diffraction data of LiInSe_2_ were collected at room temperature with a Bruker D8 ADVANCE powder diffractometer (Cu-Kα radiation) and a linear VANTEC detector. The step size of 2θ was 0.016°, and the counting time was 1.5 s per step. The 2θ range of 10–70° was measured with a 0.6 mm divergence slit, while the 2θ range of 70–140° was measured with a 2 mm divergence slit. Larger slits allow a noticeably increased intensity of high-angle peaks without the loss of resolution because the high-angle peaks are broad enough not to be affected by a more diverged beam. The esd’s σ (I_i_) of all points on patterns were calculated using intensities I_i_: σ (I_i_) = I_i_^1/2^. The intensities and obtained esd’s were further normalized: I_inorm_ = I_i_ × 0.6/(slit width), σ_norm_ (I_i_) = σ (I_i_) × 0.6/(slit width), taking into account the actual divergence slit width value, which was used to measure each particular intensity Ii, and saved in xye-type file. Such transformed powder XRD patterns can be viewed in the whole 2θ range of 10–140°, but all high-angle points have small esd’s. To prepare the powder sample for the XRD measurements, a piece of LiInSe_2_ crystal was ground with the help of mortar and pestle.

The XPS analysis was performed in ultra-high vacuum with a VGESCALAB 220i-XL system employing a monochromatic Al Kα (1486.6 eV) X-ray source. The X-ray gun was operated at 120 W, and the spectrometer pass energy was set at 20 and 100 eV for regional and survey scans, respectively. The diameter of the area under analysis was approximately 500 μm, and the probed surface layer thickness was ~5 nm. A low-energy electron flood gun was used to neutralize the surface charge buildup. The binding energies (BEs) were calibrated by fixing the saturated hydrocarbon component of the C 1s peak at 285.0 eV. The peak fitting of the overlapping Li 1s and Se 3d region was performed using the CasaXPS software package [53]. The XPS measurements were carried out for a single crystal sample.

## 3. Computation Methods

First-principles band structure and lattice dynamics properties were calculated to analyze the thermal behavior of LiInSe_2_. The calculation was carried out by CASTEP [54], a package based on the plane-wave pseudopotential density functional theory [55]. The functions developed by the Perdew, Burke, Emzerhof (PBE) [56] in the form of generalized gradient approximation (GGA) [57] were chosen to describe the exchange-correlation interaction, and optimized norm-conserving pseudopotentials [58] were adopted to model the effective interaction between the atomic cores and the valence electrons. To guarantee the precise calculation, the cutoff energy was set as 800 eV and the energy convergence tolerance for a self-consistent field calculation was set as 1 × 10^−8^ eV/atom. The intensive Monkhorst−Pack k-point meshes [59] spanning less than 0.07Å^−3^ were chosen. The phonon characters were calculated by the linear response mechanism [60]. To get the phonon modes contribution to the thermal expansion of the respective axis, the phonon frequency was first calculated on the optimized structure with the cell parameter fixed on the experimental values at 303 K. Then, the respective axis was stretched to the experimental values at 703 K, and the phonon frequency was calculated. Finally, the Gruneisen parameter (g) was calculated by the formula g = (V/ΔV) × (Δω/ω).

## 4. Results and Discussion

**Figure 2:**
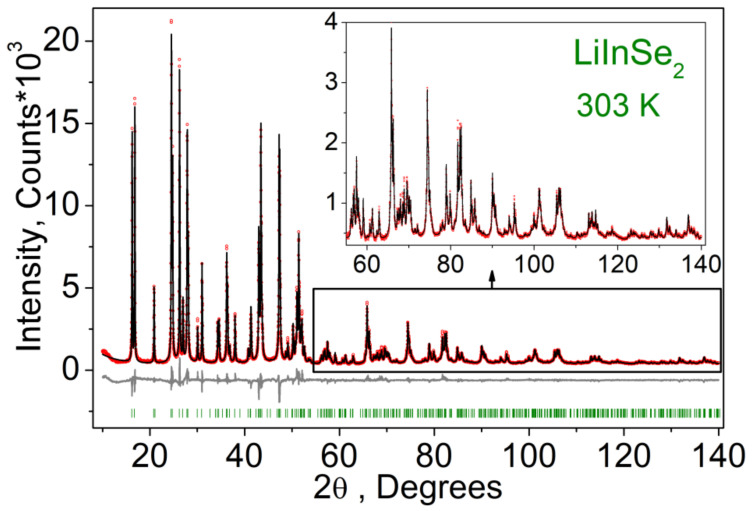
Observed, calculated (using the Rietveld method) and difference XRD patterns obtained for the LiInSe_2_ sample.

**Figure 3:**
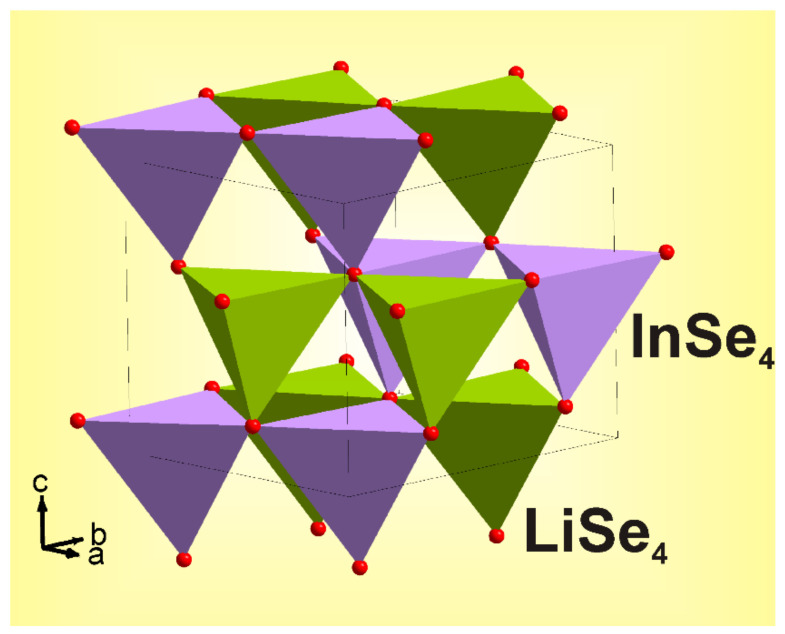
Crystal structure of LiInSe_2_. Unit cell is outlined. Lone atoms are omitted for clarity.

**Figure 4:**
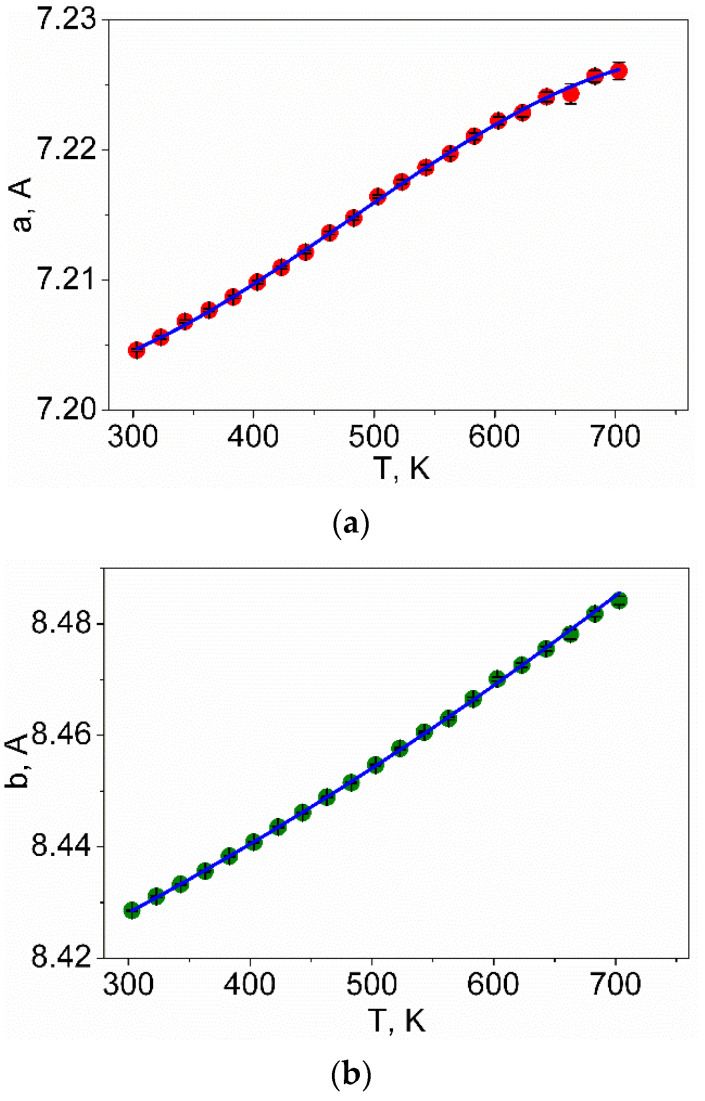

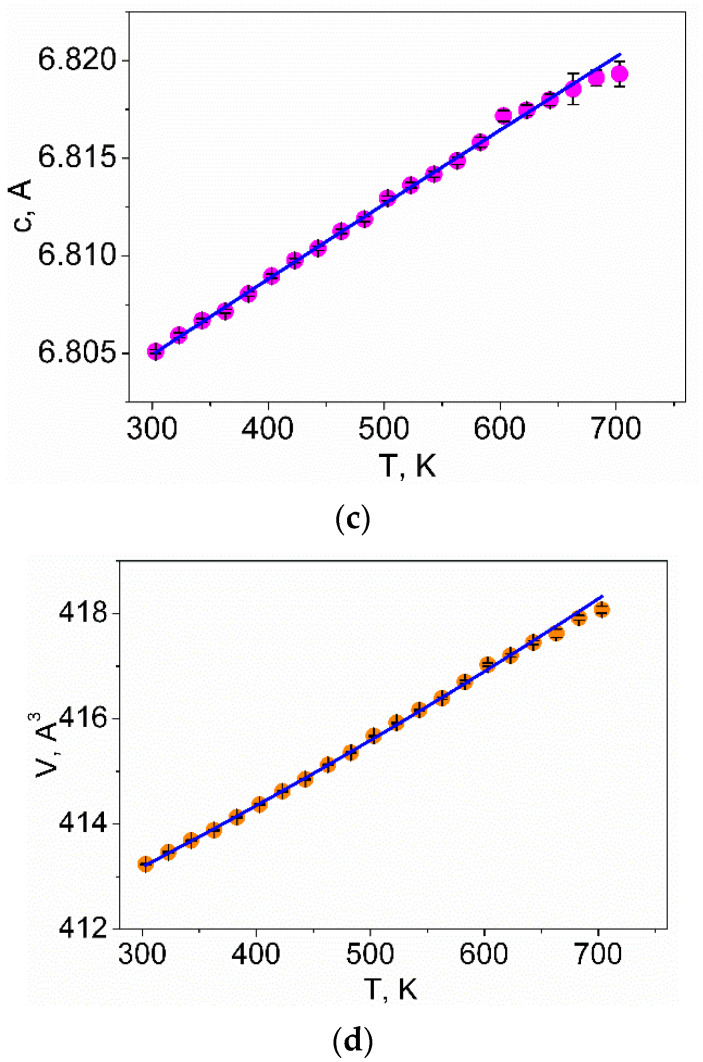
Cell parameter dependence on temperature: (**a**) *a*(T), (**b**) *b*(T), (**c**) *c*(T), and (**d**) *V*(T).

**Figure 5:**
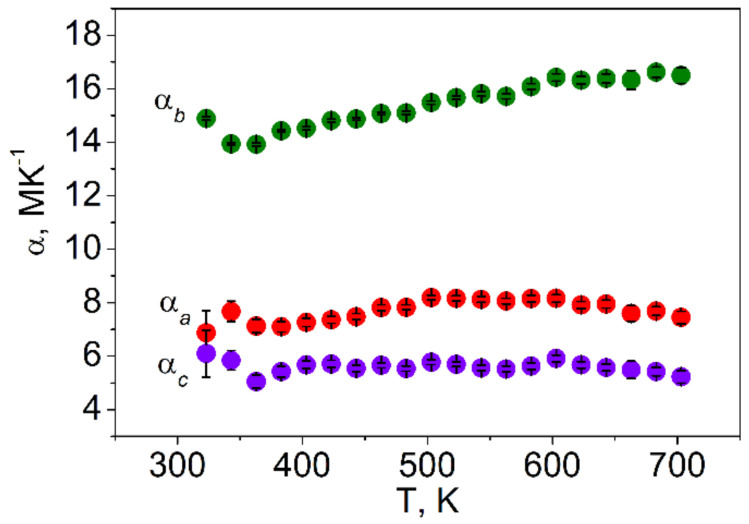
Thermal expansion coefficient dependence on temperature.

Rietveld refinement was performed by using TOPAS 4.2 [61] which accounts esd’s of each point by a special weight scheme. All peaks were indexed by an orthorhombic cell (*Pna*2_1_) with parameters close to those previously reported for LiInSe_2_ [23], and the refinement was stable resulting in low R-factors (Table 3, Figure 2). The obtained coordinates of atoms and main bond lengths are listed in Supplementary Materials, Tables S1 and S2, respectively, and the crystal structure is presented in Figure 3. To get the information on the temperature dependence of the unit cell parameters (Figure 4), 22 X-ray patterns in the 2θ range of 5–120° were collected from 303 to 723 K with 20 K step: (303, 323, 343, 363, 383, 403, 423, 443, 463, 483, 503, 523, 543, 563, 583, 603, 623, 643, 663, 683, 703, and 723 K) spending 35 min for each pattern (Figure S1). At each selected point, the temperature was fixed and controlled with precision of ±0.2 °C during the pattern measurement. The powder pattern recorded at 723 K showed a large amount of impurity and pattern intensities were not stable during the experiment. So, we stopped at this temperature because the sample decomposition was observed. Almost all peaks of all patterns were indexed by LiInSe_2_, besides small amount of impurity peaks (Figure S1) marked by asterisk (appeared at 623 K and increased their intensities under further heating) and arrow (appeared at 503 K and disappeared at 663 K). These impurity phases were not identified, but their appearance did not influence the main phase cell parameter refinement. One can see that all *a*, *b,* and *c* cell parameters and the cell volume of the main phase LiInSe_2_ increase upon heating. Therefore, LiInSe_2_ has thermal expansion behavior along all crystallographic directions. The related thermal expansion coefficients of LiInSe_2_ are reported in Table 2. As it is seen in Figure 5, the thermal expansion coefficients of LiInSe_2_ remain nearly constant with increased temperature. It should be pointed that the thermal expansion of LiInSe_2_ is strongly anisotropic, and the ratio α_b_/α_c_ is as high as ~2.9.

**Figure 6:**
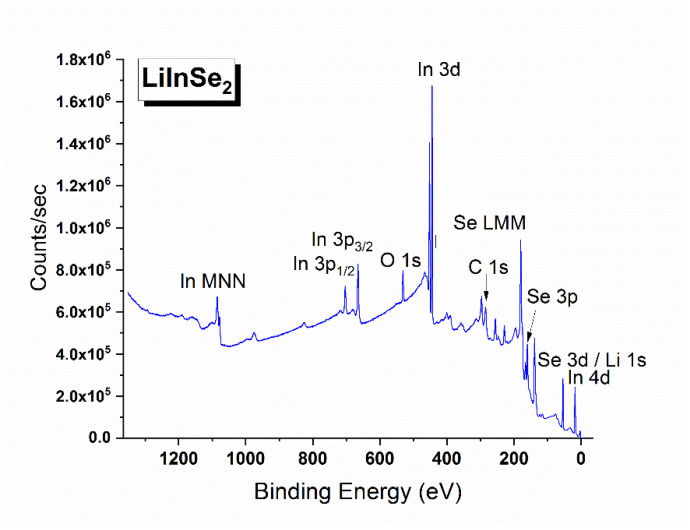
Survey X-ray photoelectron spectrum of LiInSe_2_.

**Figure 7:**
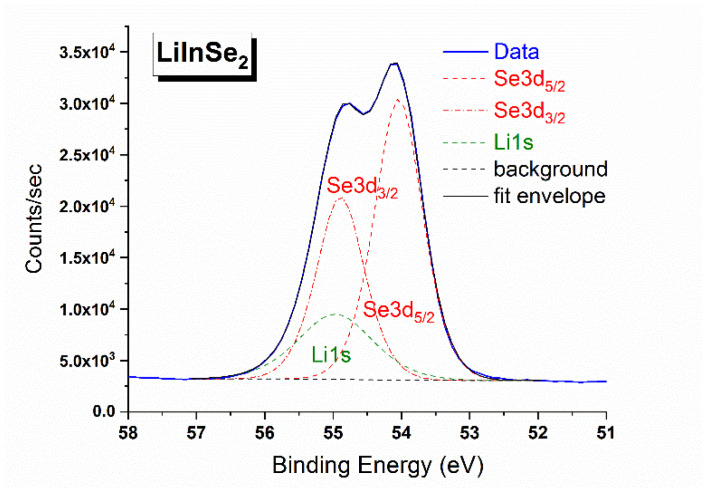
Detailed XPS spectrum of the Se 3d and Li 1s region in LiInSe_2_.

**Figure 8:**
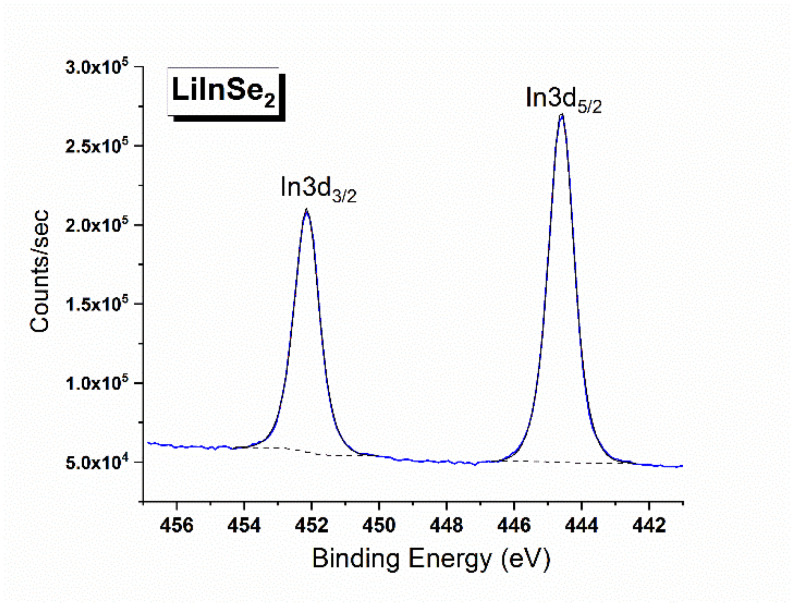
Detailed XPS spectrum of the In 3d doublet in LiInSe_2_.

The X-ray photoelectron survey spectrum recorded from LiInSe_2_ is shown in Figure 6. Besides the photoemission and Auger lines of the constituent elements, two low intensity C1s and O1s peaks were also detected. These “foreign” species are attributed to the presence of adventitious hydrocarbons and hydroxyl groups adsorbed from the ambient environment and the results of surface chemical reaction with oxygen-bearing components of the air. The detailed regional spectra were measured for the valence band and all elemental core levels detected in the survey spectrum. The overlapping spectral region of the Li 1s core level and the Se 3d doublet is shown in Figure 7. Comparing our results with those published for LiGaSe_2_ [62] and LiGa_0.5_In_0.5_Se_2_ [63], it is obvious that the spectral resolution in the current study is much better as the overlapping peaks are partially resolved in our study in contrast to the single symmetric peak observed in the previous studies. Owing to the superior resolution, it is possible for us to determine the BE values of the Li 1s, Se 3d_5/2_, and 3d_3/2_ components by employing the curve fitting procedure in CasaXPS [53]. After background subtraction, all three components were fitted with a mixed 50% Gaussian—50% Lorentzian peak. To improve the reliability of curve-fitting, the intensity ratio of the two Se spin-orbit splitting peaks, I (3d_5/2_)/I (3d_3/2_), was fixed to the theoretical value of 1.5 (i.e., ratio of the respective degeneracies 2j + 1), and the amount of the spin-orbit splitting was fixed to be 0.85 eV (based on the value reported in a high resolution XPS study of a single crystal CuInSe_2_ [64]), but no other constraints were imposed. The In 3d doublet region is presented in Figure 8. The energy positions of the constituent element core levels and Auger lines observed for LiInSe_2_ are presented in Table 4 (based on the C 1s level being fixed at 285.0 eV). In Table 5, our XPS results of LiInSe_2_ are compared to those of the representative LiMX2 crystals, with their electronic structures reported in the literature [45,62,63,65]. As mentioned above, the overlapping peaks of Li 1s and Se 3d were not resolved for LiGaSe_2_ [62] or LiGa_0.5_In_0.5_Se_2_ [63]. Therefore, the reported BE for the combined Li 1s/Se 3d peak is an overestimation for the actual value of Se 3d and underestimation of the actual value of Li 1s in [62,63]. Nevertheless, a reasonable agreement is evident, especially given the possibility of differential charging effects.

**Figure 9:**
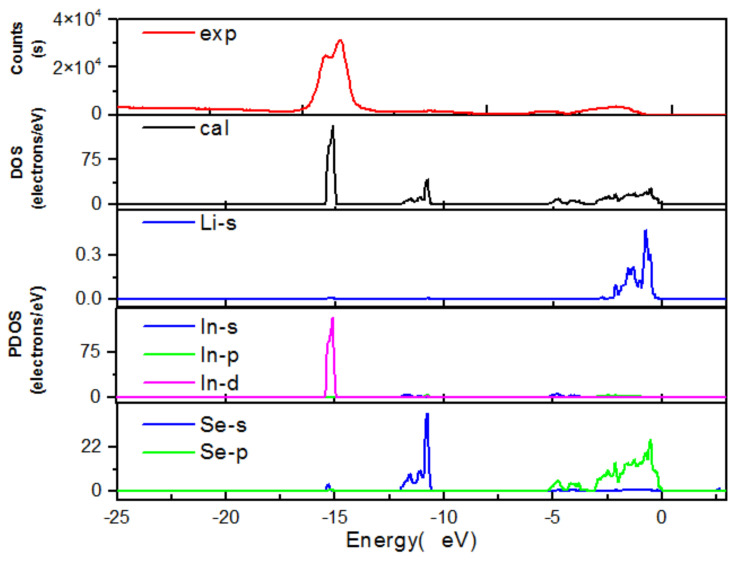
Comparison of the experimental XPS spectrum and ab initio calculated distributions of electronic states.

The experimental XPS spectrum obtained for the valence band of LiInSe_2_ is displayed in Figure 9. For comparison, the first-principles electronic density of state (DOS) and partial density of state (PDOS) projected in the electronic orbitals of the constituent atoms are also shown. Because the inner-shell electrons were excluded in the electronic structure calculations, the XPS spectrum and DOS/PDOS are exhibited only for the valence electrons. A good agreement is reached between the experimental and calculated spectra, especially for the energy position of the respective electronic orbitals, indicating the validity of the plane-wave pseudopotential method for the studied crystal. The measured spectrum is slightly broadened compared to the calculated results, which might be attributed to the thermal effect, as well as instrumental broadening associated with the experiment. From the calculated PDOS, the feature of anionic crystal for LiInSe_2_ can be clearly deduced: almost all the electronic orbitals are strongly localized and the hybridization with the others is very small.

**Figure 10:**
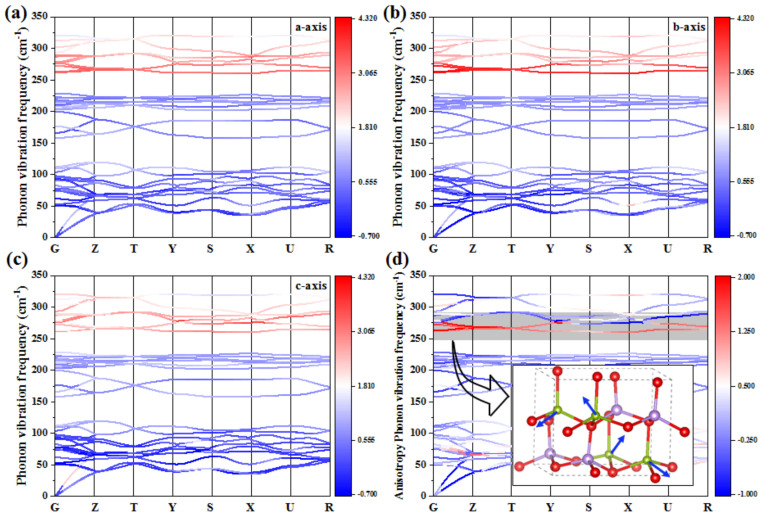
(**a**–**c**) The phonon dispersion of the structure with the perturbation of axes *a*, *b*, and *c*, respectively, in which the Grüneisen values of different vibration mode were presented by the color from blue to red. (**d**) The phonon dispersion of the structure at 303 K, in which the subtracted Grüneisen values of *a*-axis and *c*-axis were presented by the color from blue to red. The inset in (**d**) is the graphic vibration mode of around 260 cm^−1^, and the vibration vectors are shown as blue arrows.

The anharmonicity of lattice vibration phonon is the principal source of thermal expansion of solids. To shed light on the mechanism of the thermal expansion anisotropy in LiInSe_2_, Grüneisen constant, the parameter that characterizes the phonon anharmonicity was calculated. Accordingly, no imaginary frequency is observed in the phonon spectrum, which demonstrates the dynamical stability of LiInSe_2_. According to the Grüneisen–constant-colored phonon dispersion, one can see that for all the three axes, the phonon modes in the range of 250~300 cm^−1^ have the maximum Grüneisen constants (Figure 10a–c), i.e., maximum phonon anharmonicity. This suggests that these phonon modes make the major contribution to the thermal expansion along all the three modes. By subtracting the Grüneisen constants along the maximum (*c*-axis) and minimum (*a*-axis) thermal expansion coefficient, the phonon modes accounting for the thermal expansion anisotropy can be obtained. Accordingly, as shown in Figure 10d, the modes with the largest Grüneisen constant difference are mainly located around 260 cm^−1^. Atomic vibrational assignment reveals that these modes mainly arise from the vibration of lithium atoms, and this demonstrates that lithium atoms play a key role in determining the thermal expansion anisotropy in LiInSe_2_.

## 5. Conclusions

In this study, a big-sized high-quality LiInSe_2_ crystal was grown by the developed Bridgman–Stockbarger method. The anisotropic thermal expansion behavior of the LiInSe_2_ crystal was measured for the first time. Combining the experimental characterization and theoretical calculations, the huge thermal expansion anisotropy was attributed to the vibration of lithium atoms. In addition, the electronic structure of LiInSe_2_ crystal was measured by XPS and the recorded valence band is in a good agreement with the theoretical electronic density of states. Moreover, the Grüneisen parameters were also calculated in the theory to reveal the dominant source of the thermal expansion anisotropy. These results indicate that LiInSe_2_, besides its well-known pronounced linear and nonlinear optical properties in the IR spectral range, possesses specific structural effects.

## Figures and Tables

**Table 1:** Structural types known in compounds LiMX_2_ (M = Al, In, Ga; X = S, Se, Te).

M	S	Se	Te
In	*P*21*nb* (*Pna*2_1_) [19]	*Pna*2_1_ [23]	*I*-42*d* [24]
Ga	*Pna*2_1_ [20,21]	*Pna*2_1_ [21,22]	*I*-42*d* [21]
Al		*Pna*2_1_ [25]	*I*-42*d* and *P*3*m*1 [25]

**Table 2:** Thermal expansion coefficients of LiMT_2_ (M = Ga, In; T = Se, Te) crystals.

Compound	Type	α_a_ MK^−1^	α_b_ MK^−1^	α_c_ MK^−1^	α_V_ MK^−1^	Reference
LiGaTe_2_	II	19.1	19.1	−8.6	29.4	[45]
LiGa_0_._55_In_0_._45_Te_2_	II	18.9	18.9	−5.7	32.3	[46]
LiGa_0_._54_In_0_._46_S_2_	I	11.7	15.8	12.7		[44]
LiInS_2_	I	8.9	16.1	6.6		[2]
LiInSe_2_	I	11.5 ± 1.7	20.4 ± 2.4	8.9 ± 2.4		[43]
LiInSe_2_	I	8.1 (1)	16.1 (2)	5.64 (6)	29.9 (3)	This work

**Table 3:** Main parameters of processing and refinement of the LiInSe_2_ sample.

Compound	LiInSe_2_
Sp. Gr.	*Pna*2_1_
*a*, Å	7.20442 (7)
*b*, Å	8.42826 (8)
*c*, Å	6.80491 (6)
*V*, Å^3^	413.199 (7)
*Z*	4
*2θ*-range, °	10–140
*R_wp_*, %	5.63
*R_p_*, %	4.71
*R_exp_*, %	2.67
*χ* ^2^	2.11
*R_B_*, %	2.16

**Table 4:** Core level binding energies and Auger lines in LiInSe_2_.

Line	Binding Energy, eV
In 4d_5/2_	18.06
In 4d_3/2_	18.93
Se 3d_5/2_	54.04
Se 3d_3/2_	54.89
Li 1s	54.97
Se L3M45M45	179.95
C 1s	285.0 (fixed)
O 1s	531.46
In 3d_5/2_	444.60
In 3d_3/2_	452.15
In M4N45N45	1078.70

**Table 5:** BE values of representative core levels measured in LiMX_2_ materials.

Crystal	Ga 3d	Li 1s	In 3d_5/2_	S 2p	Se 3d_5/2_	Te 3d_5/2_	Ref.
LiGaS_2_	20.1	55.3	-	162.0	-	-	[65]
LiGaSe_2_	19.72	54.23 *	-	-	54.23 *	-	[62]
LiGaTe_2_	19.3	55.0	-	-	-	572.3	[45]
LiGa_0_._5_In_0_._5_Se_2_	18.26 **	54.23 *	444.82	-	54.23 *	-	[63]
LiInSe_2_	-	54.97	444.60	-	54.04	-	This study

* superposition (Li 1s + Se 3d). ** superposition (Ga 3d + In 4d). Note that values reported in [45] were shifted by +0.2 eV due to their C 1s level being fixed at 284.8 eV, and values in [62,63,65] were shifted by +0.4 eV because their C 1s level was fixed at 284.6 eV.

## Data Availability

Data are available on request to authors.

## Supplementary Materials

The following supporting information can be downloaded at: https://www.mdpi.com/article/10.3390/molecules27165078/s1, Figure S1: X-ray patterns collected from 303 to 703 K. The major impurity peaks appeared under heating are marked by asterisk and arrow; Table S1: Fractional atomic coordinates and isotropic displacement parameters (Å^2^) of LiInSe_2_; Table S2: Main bond lengths (Å) of LiInSe_2_.

## Sample Availability

Samples of the compounds are not available from the authors.
