# Supplementary material for: Anisotropic Thermal Expansion and Electronic Structure of LiInSe2
Source: Molecules. 2022 Aug 10;27(16):5078. doi: 10.3390/molecules27165078 (PMC9413981; doi:10.3390/molecules27165078)
Supplement: Supplementary file 1 [file molecules-27-05078-s001.zip › molecules-1816259-supplementary.pdf]

Supplementary Materials

Anisotropic Thermal Expansion and Electronic Structure of $LiInSe_2$

Victor V. Atuchin [1,2,3,4,5,*], Ludmila I. Isaenko [6,7], Sergei I. Lobanov [6,7], Alina A. Goloshumova [6,7], Maxim S. Molokeev [8,9,10], Zhaoming Zhang [11], Xingyu Zhang [12,13], Xingxing Jiang [12] and Zheshuai Lin [12,13]

[1] Laboratory of Optical Materials and Structures, Institute of Semiconductor Physics, SB RAS, Novosibirsk 630090, Russia
[2] Department of Applied Physics, Novosibirsk State University, Novosibirsk 630090, Russia
[3] Research and Development Department, Kemerovo State University, Kemerovo 650000, Russia
[4] Department of Industrial Machinery Design, Novosibirsk State Technical University, Novosibirsk 630073, Russia
[5] R&D Center "Advanced Electronic Technologies", Tomsk State University, Tomsk 634034, Russia
[6] Laboratory of Crystal Growth, Sobolev Institute of Geology and Mineralogy, SB RAS, Novosibirsk 630090, Russia
[7] Laboratory of Functional Materials, Novosibirsk State University, Novosibirsk 630090, Russia
[8] Laboratory of Crystal Physics, Kirensky Institute of Physics, Federal Research Center KSC SB RAS, Krasnoyarsk 660036, Russia
[9] Institute of Engineering Physics and Radioelectronic, Siberian Federal University, Krasnoyarsk 660041, Russia
[10] Department of Physics, Far Eastern State Transport University, Khabarovsk 680021, Russia
[11] Australian Science and Technology Organisation (ANSTO), Lucas Heights, NSW 2234, Australia
[12] Functional Crystals Laboratory, Technical Institute of Physics and Chemistry, Chinese Academy of Sciences, Beijing 100190, China
[13] University of the Chinese Academy of Sciences, Beijing 100049, China
* Correspondence: atuchin@isp.nsc.ru; Tel.: +7-(383)-3308889

Table S1. Fractional atomic coordinates and isotropic displacement parameters ($Å^2$) of $LiInSe_2$.

	x	***y***	***z***	$\boldsymbol{B_{iso}}$
In	0.0774 (2)	0.1259 (9)	0	1.39 (6)
Li	0.077 (5)	0.62 (2)	0.01 (2)	1.7 (9)
Se1	0.0812 (3)	0.127 (2)	-0.3795 (8)	1.13 (6)
Se2	0.4134 (4)	0.122 (2)	0.1275 (8)	1.13 (6)

Table S2. Main bond lengths (Å) of $LiInSe_2$.

In—Se1	2.583 (5)	Li—Se1^{iii}	2.5 (2)
In—Se1^{i}	2.55 (1)	Li—Se1^{iv}	2.58 (5)
In—Se2	2.572 (3)	Li—Se2^{v}	2.6 (1)
In—Se2^{ii}	2.58 (1)	Li—Se2^{ii}	2.5 (2)

Symmetry codes: (i) -*x*, -*y*, *z*+1/2; (ii) *x*-1/2, -*y*+1/2, *z*; (iii) -*x*, -*y*+1, *z*+1/2; (iv) -*x*+1/2, *y*+1/2, *z*+1/2; (v) -*x*+1/2, *y*+1/2, *z*-1/2.

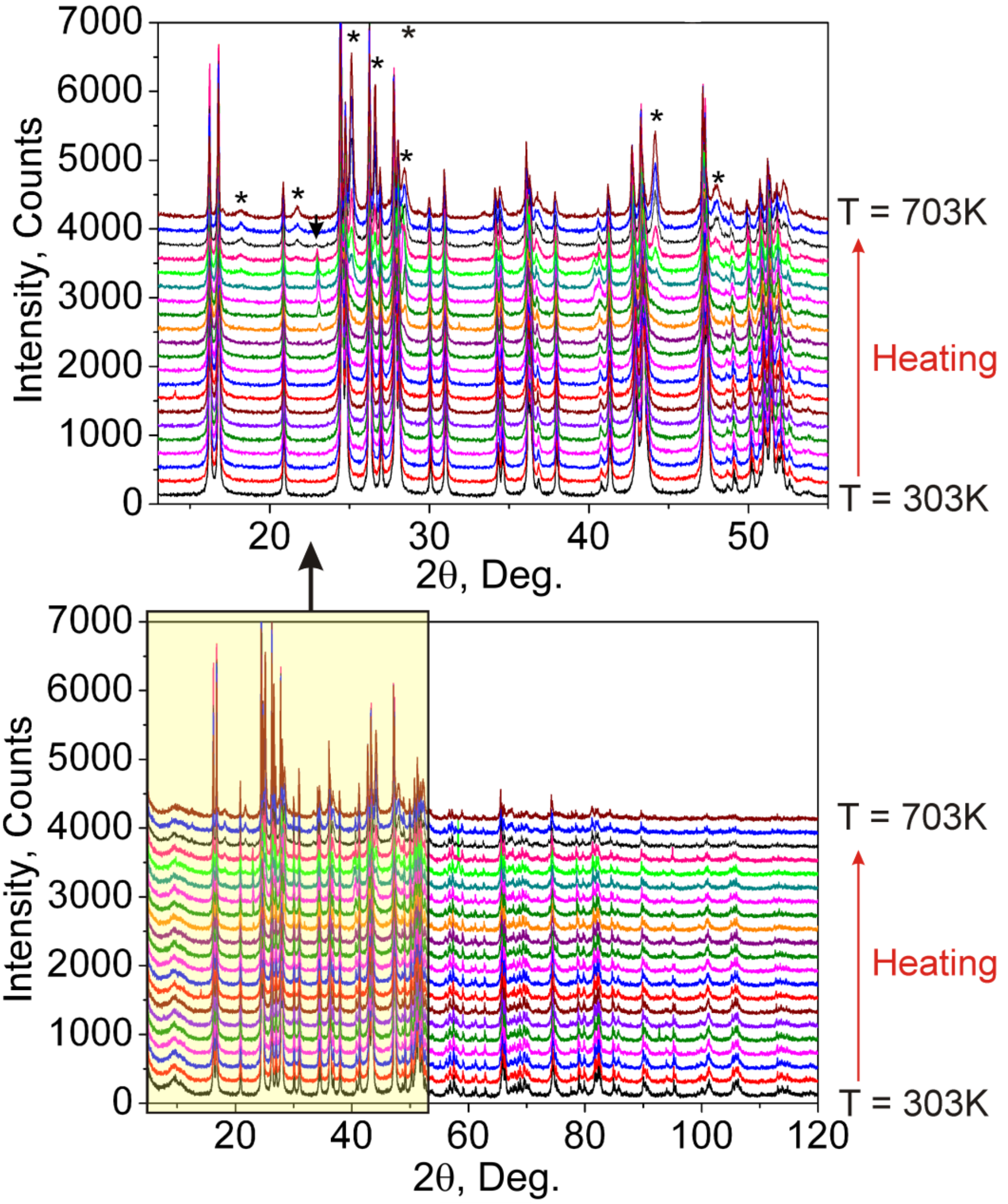

Figure S1. X-ray patterns collected from 303 to 703 K. The major impurity peaks appeared under heating are marked by asterisk and arrow.
